# Does the prenatal bisphenol A exposure alter DNA methylation levels in the mouse hippocampus?: An analysis using a high-sensitivity methylome technique

**DOI:** 10.1186/s41021-018-0099-y

**Published:** 2018-06-04

**Authors:** Toshiki Aiba, Toshiyuki Saito, Akiko Hayashi, Shinji Sato, Harunobu Yunokawa, Toru Maruyama, Wataru Fujibuchi, Seiichiroh Ohsako

**Affiliations:** 1 Laboratory of Environmental Health Science, Center for Disease Biology and Integrative Medicine, Graduate School of Medicine, The University of Tokyo, 7-3-1 Hongo, Bunkyo-ku, Tokyo, 113-8654 Japan; 2 Department of Radiation Effects Research, National Institutes for Quantum and Radiological Science and Technology, 4-9-1 Anagawa, Inage-ku, Chiba, 263-8555 Japan; 3 Maze, Inc, 1-2-17 Sennincho, Hachioji-shi, Tokyo, 193-0835 Japan; 4 Center for iPS Cell Research and Application, Kyoto University, 53 Kawahara-cho, Shogoin, Sakyo-ku, Kyoto, 606-8507 Japan

**Keywords:** Bisphenol A, Hippocampus, DNA methylation, Epigenetics

## Abstract

**Background:**

There is still considerable debate about the effects of exposure to bisphenol A (BPA) an endocrine disrupter at low doses. Recently, many studies using animal models have shown that prenatal BPA exposure induces behavioral and neuronal disorders due to epigenetic changes in the brain. However, striking evidence of epigenomic changes has to be shown.

**Methods:**

To investigate whether low-dose BPA exposure in the fetal stage can alter CpG methylation levels in the central nervous system, the hippocampus of the inbred C57BL/6 J mouse as the target tissue was collected to detect alterations in CpG methylation levels using a highly sensitive method of genome-wide DNA methylation analysis, methylated site display–amplified fragment length polymorphism (MSD-AFLP).

**Results:**

BPA showed the sex-hormone like effects on male reproductive organs. Although we examined the methylation levels of 43,840 CpG sites in the control and BPA (200 μg/kg/day)-treated group (6 mice per group), we found no statistically significant changes in methylation levels in any CpG sites.

**Conclusions:**

At least under the experimental condition in this study, it is considered that the effect of low-dose BPA exposure during the fetal stage on hippocampal DNA methylation levels is extremely small.

**Electronic supplementary material:**

The online version of this article (10.1186/s41021-018-0099-y) contains supplementary material, which is available to authorized users.

## Background

Bisphenol A (BPA), an endocrine disruptor, has still been the focus of attention of scientists in the field of environmental health since the last century. Even today, many researchers in field of hygiene and environmental health science are investigating exposure level in human population [1, 2], and reporting studies focusing on mechanistic aspects [3–5]. An initial study found that BPA monomer released from a polycarbonate dish has an estrogenic activity in a cell culture study [6]. Subsequently, in experimental animal studies, exposure of pregnant animals to BPA at a low doses (2–20 μg/kg) altered the development of male reproductive organs such as the prostate [7–9]. It was also reported that the sperm count decreased in adult male rats even at a low dose of BPA exposure (20 μg/kg/day) [9]. This dose was comparable to the maximum exposure levels (375–857 ng/kg/day) for infants [10]. Subsequently, similar experiments on a low-dose of BPA exposure were performed by research groups associated with some industrial companies [11–13]. However, they failed to reproduce the above described results. Furthermore, the rat two-generation and three-generation reproductive toxicity studies carried out by independent groups did not detect changes in the reproductive organs by BPA diet throughout the experiments [14, 15]. These inconsistent conclusions brought the story of BPA into complex.

After a while, with the focus of studies shifting away from reproductive toxicity, a number of studies started to show that low-dose BPA exposure in the perinatal period affected behavioral and brain development [16–18]. In particular, the effects of low-dose BPA exposure on the hippocampus were observed in many studies. BPA was found to decreased hippocampal spine number as induced by estrogen in the African green monkey (*Chlorocebus aethiops sabaeus*) [19], inhibit acetylcholine production in mice [20], and reduce the spine density in the mouse CA1 region [21], suggesting that perinatal BPA exposure may cause developmental disturbance.

Such effects of BPA can be regarded as the Developmental Origins of Health and Disease (DOHaD), that is fetal/neonatal environmental factors are the causes of diseases after maturation [22–24]. It is hypothesized that exposure to environmental factors changes epigenomic states, such as DNA methylation levels, which remain after birth [25, 26]. Furthermore, many challenging studies reported that gestational exposure to environmental chemicals, such as vinclozolin and arsenite, causes in epigenetic transgenerational changes in rodents [27–29]. Now the chemical-induced epigenomic alteration is extremely controversial issue not only in DOHaD studies but also in general biology concerning evolution [30]. In our series of environmental epigenetics studies, we found that a single and low dose of 2,3,7,8-tetrachlorodibenzo-*p*-dioxin induced hypomethylation of CpGs in *Cyp1a1* gene promoter region in mouse liver [31]. This hypomethylation was induced by adulthood exposure, but also by in utero exposure [32]. Thus, we convinced that a low level of environmental pollutants causes the alternation of DNA methylation status.

Indeed, several animal studies have shown that prenatal exposure to BPA causes epigenetic changes [33–38]. However, there are only a few studies using a sufficient number of inbred animals for comparison and/or by genome-wide DNA methylation analysis. Recently, we have developed a technique called methylated site display-amplified fragment length polymorphism (MSD-AFLP) analysis, which is a sensitive and affordable method of CpG methylation profile analysis [39]. MSD-AFLP analysis is cost-effective because multiple samples can be analyzed at the same time. MSD-AFLP analysis is also a very sensitive technique because it can detect slight changes in DNA methylation levels. Therefore, in this study, a low-dose BPA (200 μg/kg/day) was administered to pregnant B57BL/6 J mice and the changes of DNA methylation level in the hippocampus of offspring were detected by MSD-AFLP analysis.

## Methods

### Reagents

The reagents and materials used in this study were as follows. BPA and corn oil were purchased from Wako Pure Chemical Industries (Osaka, Japan). T4 DNA ligase and restriction enzymes *Hpa* II, *Msp* I, *Sbf* I were from New England Biolabs (MA, USA). AllPrep DNA/RNA mini kit was from Qiagen (Hilden, Germany). Oligonucleotides were from Operon (Alameda, Calif., USA) and streptavidin-coated magnetic beads (Dynabeads M-280 Streptavidin) were from Dynal (Oslo, Norway). TITANIUM Taq DNA polymerase was from Takara Bio (Kusatsu, Japan) and LightCycler® 480 SYBR Green I Master was from Roche (Diagnostics GmbH, Mannheim, Germany). POP-7™ Polymer, GeneScan™ 500 LIZ® Size Standard and BigDye® Terminator v3.1 Cycle Sequencing Kit were from ThermoFisher Scientific Inc., (San Diego, CA, USA).

### Animals and treatments

**Fig. 1:**
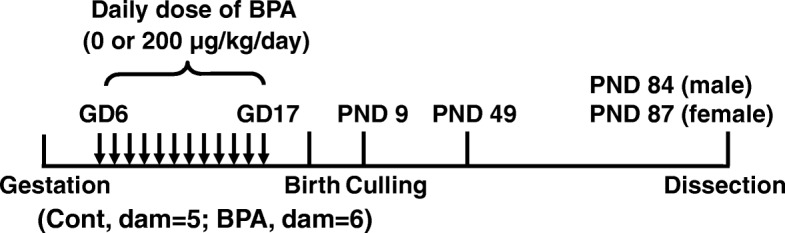
Experimental design of prenatal BPA exposure to mice. Vehicle (corn oil) or BPA was orally administered to female C57BL/6 J mice during gestation at a dose of 200 μg/kg/day daily by gavage from GD 6 to GD 17. Pups were eliminated to be of the same number per mother on PND 9. Body weight and AGD were measured from PND 0 to PND 49. Male (PND 84) and female (PND 87) mice were sacrificed by cervical dislocation

Pregnant C57BL/6 J mice were purchased from CLEA Japan (Tokyo, Japan). The mice were bred at temperature of 23 °C, in 12/12 h light/dark cycle (animal facility, 08: 00–20: 00). Laboratory rodent feed (Lab MR stock, Nosan, Yokohama, Japan) and distilled water were provided ad libitum. BPA in vehicle (corn oil) or vehicle only was orally administered for 12 consecutive days at a dose of 200 μg/kg/day from the 6th to 17th days of gestation (GD); (Fig. 1). Pup mice were culled to 5 males and 3 females on postnatal day 9 (PND 9) and maintained under the same conditions. Body weight and anogenital distance (AGD) were measured from PND 0 to PND 49. Male (PND 84) and female (PND 87) mice were killed by cervical dislocation. Body weight and weights of some tissues [liver, heart, kidney, urogenital complex (UGC), testes, and uterus] were measured. The hippocampus was dissected on ice, stored at − 80 °C, and used for DNA methylation analysis. The animal experiment protocol was approved by the Animal Care and Use Committee of the University of Tokyo (I-P11–015).

### MSD-AFLP

MSD-AFLP analysis was performed in accordance with our previous report [39]. Briefly, genomic DNA was isolated from the male pup hippocampus using the AllPrep DNA/RNA Mini Kit. Genomic DNA (100 ng) was digested with the primary restriction enzymes *Sbf* I and *Msp* I, and then ligated using adaptor A (Upper, b-TCC GAC TGG TAT CAA CGC AGA GTA CTA GAG TTG CA; Lower, p-ACT CTA GTA CTC TGC GTT GAT ACC AGT CGG A; here, b indicates 5′-biotiylation and p, 5′-phospholation). The ligated DNA fragment was digested with methylation-sensitive *Hpa* II. After the ligation reaction of adaptor B (Upper, AAT GGC TAC ACG AAC TCG GTT CAT GAC C; Lower, p-CGT GTC ATG AAC CGA GTT CGT GTA GCC ATT), pre-PCR was performed using adapter-specific primers (Forward, AAT GGC TAC ACG AAC TCG GTT CAT GAC ACG G; Reverse, TCC GAC TGG TAT CAA CGC AGA) to amplify only the genomic DNA fragments that have methylated CpG at the end. The pre-PCR amplicon (MSD library) was subjected to selective PCR using 256 primer sets (Forward, f-AAT GGC TAC ACG AAC TCG GTT CAT GAC AII INN; Reverse, AGA GTA CTA GAG TTG CAG GNN; here, f means 5′-6-carboxyfluorescein (6-FAM) conjugation; I, inosine). The product of selective PCR was electrophoresed using a capillary sequencer to obtain an AFLP chart.

### GFDB analysis

The genomic position was predicted from the AFLP peak chart using the Genome DNA Fragment Database (GFDB) system. GFDB can simulate the MSD-AFLP procedure of genomic DNA cleavage with any restriction enzyme or any selective PCR [39].

### MSRE–PCR

Methylation-sensitive restriction-enzyme-dependent PCR (MSRE-PCR) analysis of two CpGs was carried out as described in our previous study {Amenya, 2016 #432}. The following primers were used in this study: Chr 4: 35339023 (Forward, TCA CTC TTC ACC TGC AGG AAG; Reverse, GCT GTC ACT CTG TGC TCT TCT) and Chr X: 74707008 (Forward, CTG CTT TGC TGC TCA GAG TTT; Reverse, CCC GAG TCC TGA GAT TAA AGG). Purified genomic DNA (100 ng) was divided into two portions. One portion was digested with *Hpa* II and the other was not digested. Both were subjected to quantitative PCR analysis using Light Cycler 480 and the methylation level of CpG was calculated as the ratio of target copy number from nondigested DNA to that from *Hpa* II-digested DNA.

### Statistical analysis

The significance of differences in the tissue weight and MSRE-PCR measurement analyzed by Student’s *t-test*. *p*-value less than 0.05 was considered statistically significant. The significance of differences in methylation level between groups was analyzed by adjusted *p*-value (*q*-value, false discovery rate (FDR)) calculated with Benjamini–Hochberg (BH) correction after Student’s *t-test* using R statistical software. Statistical probabilities of FDR less than 0.05 were considered significant. Hierarchical clustering analysis (HCA) and principal component analysis (PCA) of methylation patterns were performed using Euclidean distance, and the unweighted pair grouping method using arithmetic mean (UPGMA) was carried out. The Kyoto Encyclopedia Genes and Genome (KEGG) enrichment analysis was performed using Gene Set Enrichment Analysis (GSEA) software.

## Results

### Reproductive outcome

Prenatal BPA exposure did not significantly affect litter size or sex ratio compared with controls (Additional file 1: Table S1). In both sexes, the BPA-exposed group showed no effect on body weight or AGD from birth to PND 49 (Additional file 1: Figure S1). However, BPA exposure significantly decreased the UGC weight of male on PND 84 (Table 1). There were no significant differences in other tissues.

**Table 1:** Effects of prenatal BPA exposure on tissue weight of mice at dissection^a^

	Control		BPA	
	Male (*n* = 24)	Female (*n* = 14)	Male (*n* = 19)	Female (*n* = 12)
Liver	4.340 ± 0.063	5.703 ± 0.284	4.231 ± 0.066	5.183 ± 0.196
UGC	1.030 ± 0.015	N/A	0.941 ± 0.026*	N/A
Testis	0.764 ± 0.012	N/A	0.743 ± 0.014	N/A
Uterus	N/A	0.686 ± 0.036	N/A	0.567 ± 0.083
Heart	0.576 ± 0.014	0.514 ± 0.011	0.573 ± 0.013	0.501 ± 0.012
kidney	1.138 ± 0.017	1.158 ± 0.019	1.138 ± 0.014	1.146 ± 0.017

^a^, Tissue weight data was expressed as percentage per body weight

N/A, not available

Data were expressed as means ± SE. Statistically significant difference between means from control group was analyzed by *t-test* (*: *p* < 0.05)

### Overall effect of BPA exposure on CpG methylation

**Fig. 2:**
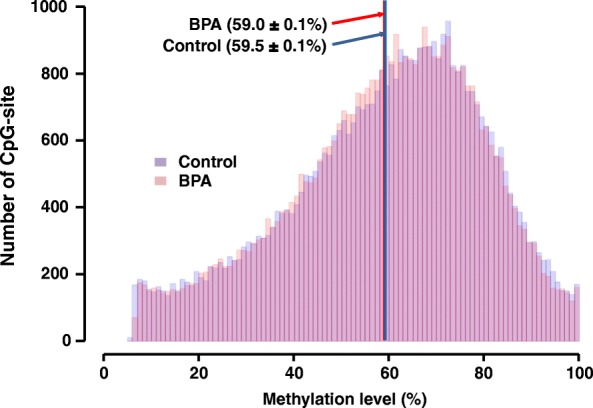
Histogram of methylation levels of all CpGs detected by MSD-AFLP analysis. Blue and red lines indicate the mean methylation levels in the control and BPA-exposed groups

**Fig. 3:**
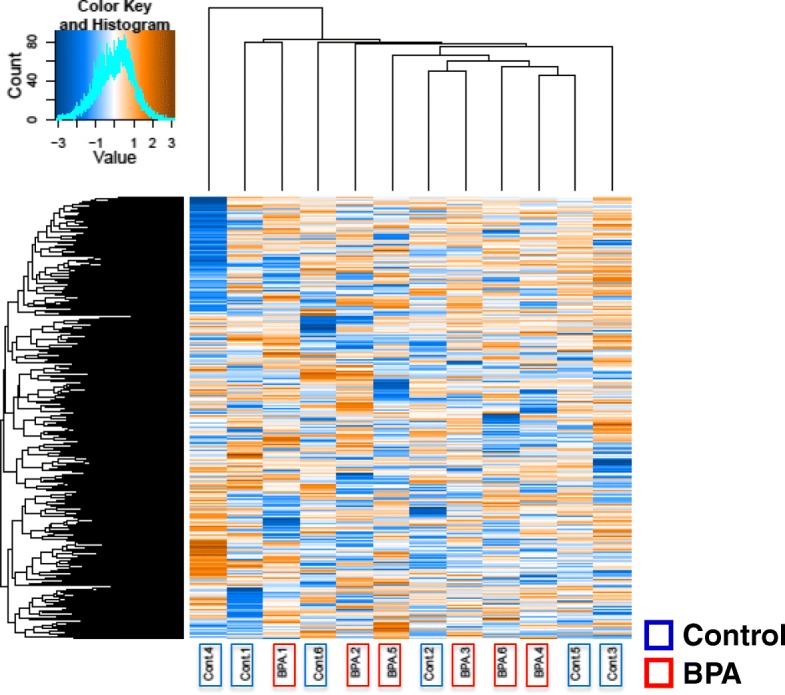
Hierarchical clustering analysis (HCA) of the effects of BPA. HCA of normalized methylation patterns of each sample using Euclidean distance and weighted pair grouping method using arithmetic mean (UPGMA)

**Fig. 4:**
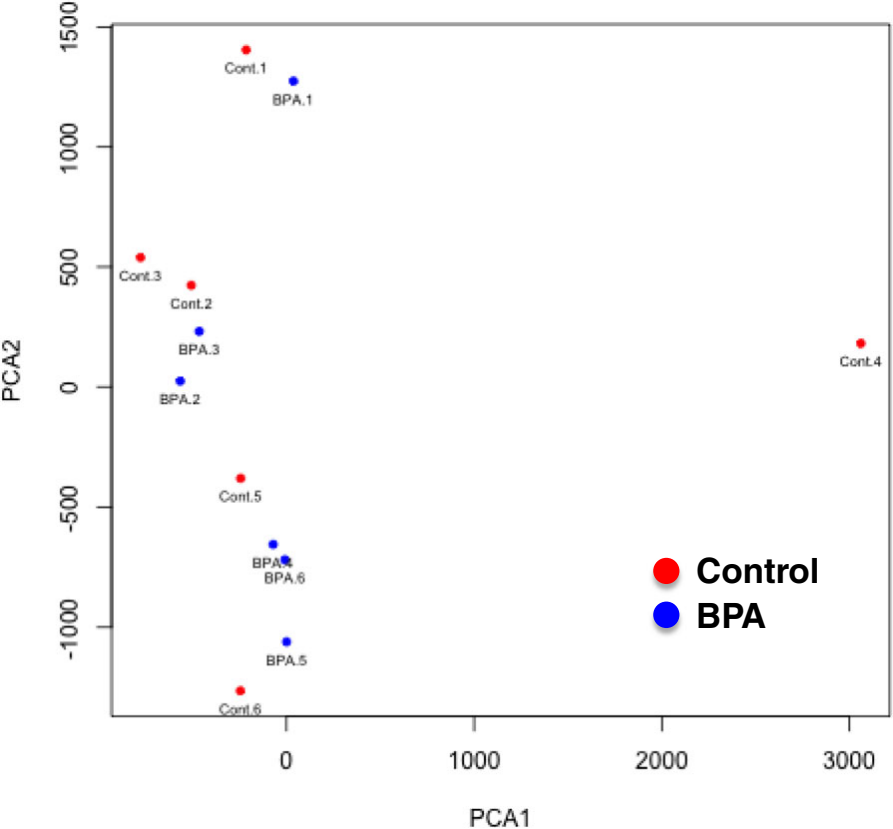
Principal component analysis (PCA) using MSD-AFLP data. Control, red circle (*n* = 6); BPA, blue circle (n = 6). PCA showed large variances of both control and BPA groups with no clusters

We investigated the effects of prenatal BPA exposure on the CpG methylation profile of the hippocampus in male pups by MSD–AFLP analysis (Additional file 1: Figure S2). A total of 43,840 CpG data were obtained by using 256 selective primer sets. The mean methylation levels were 59.5 ± 0.1% in the control group and 59.0 ± 0.1% in the BPA-exposed group (*p* = 0.22); (Fig. 2). There was no significant difference between control and BPA samples in the HCA (Fig. 3). Moreover, PCA showed no clusters and large variances of both control and BPA groups (Fig. 4). These observations suggest that prenatal BPA exposure hardly affects DNA methylation in the mouse hippocampus.

### Statistical analysis for possibility of BPA-induced change in CpG methylation

**Fig. 5:**
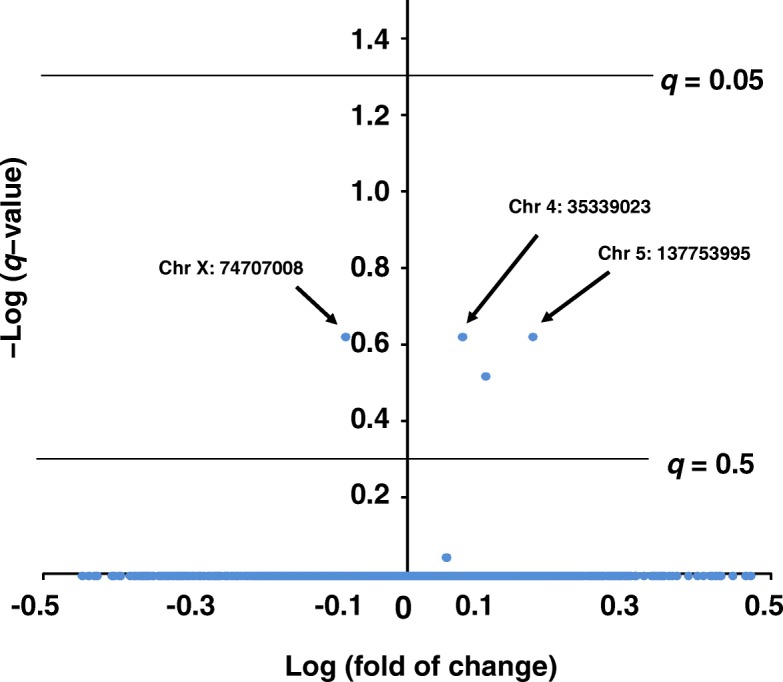
Effects of BPA on CpG methylation profile in hippocampus. Volcanic plot showing the difference in methylation level between control group and BPA-exposed group. The logarithmic value of the fold of change (BPA/control) is presented in the x-axis, and the logarithmic value of the q-value is presented in the y-axis. Three CpGs which show less than 0.24 of q-value are indicated by arrows

We analyzed the significance of differences in methylation level between groups by *q*-value calculated with BH correction after Student’s *t-test* using all CpGs data determined by MSD-AFLP to explore possibility of BPA-induced changes. However, we detected no CpGs showing less than 0.05 of *q*-value of FDR. Figure 5 is a volcanic plot of *q*-value of FDR and fold of changes by BPA exposure. We found only three CpGs shows the lowest *q*-value of FDR (*q* = 0.24, −log(*q*-value) = 0.620) out of 43,840 CpGs. The genomic positions of three CpGs predicted by using the GFDB system were shown in Table 2. Using the genomic DNA samples, in order to examine if fold of changes detected by MSD-AFLP are significant, we performed MSRE-PCR analysis. Since another *Hpa* II site is located on immediately nearby the CpG (Chr 5: 137753995), it was impossible to design primers that are amplifiable in MSRE-qPCR. Therefore, we examined the remaining only two CpGs (Chr 4: 35339023 and Chr X: 74707008). Although similar trends of difference between control and BPA in both CpGs shown in MSD-AFLP analysis were detected, neither of the two CpGs showed significant difference in the MSRE-PCR analysis (Additional file 1: Figure S3).

**Table 2:** The genomic positions of three CpGs showing minimum *q*-value predicted by using GFDB

Chr.	Position^a^	Gene name	Methylation level (%)		log ratio^b^	*q*-value
			Cont (n = 6)	BPA (n = 6)		
5	137,753,995	Trip6	18.21 ± 0.79	26.85 ± 0.67	0.17	0.24
4	35,339,023	9.3kbp up stream of RP23-307 M2.4	29.36 ± 0.34	34.67 ± 0.52	0.07	0.24
X	74,707,008	4.4kbp down stream of RP23-238B14.1	80.53 ± 1.38	65.66 ± 1.35	−0.09	0.24

^a^, Chromosomal nucleotide position of methylated cytosine

^b^, The ratio means fold of change (BPA/control)

### The KEGG enrichment analysis of CpG methylation level

The enrichment analysis of CpG methylation level on the intragenic region and promoter region (5 kb upstream from TSS) was performed to examine if statistical difference is detected in KEGG pathway (Additional file 1: Table S2). The hypermethylated CpGs in BPA exposure were tended to highly enrich in the pathway of N-Glycan Biosynthesis (NES = 1.600; FDR = 0.77). On the other hands, the hypomethylated CpGs in BPA exposure were tended to highly enrich in the pathway of Histidine Metabolism (NES = 1.602; FDR = 0.917). However, the minimum FDR was 0.776, no pathway with significantly different methylation level was detected according to the criteria FDR < 0.05.

## Discussion

In this study we investigated whether prenatal low-dose BPA exposure causes epigenomic changes in the cerebral hippocampus. Our study showed that the weight of the male UGC consisting of three tissues, i.e., the prostate, seminal vesicle, and urethra, was significantly reduced. Although this observation is in contrast to a previous report on prostate development [8], our experiment also showed sex-hormone-like activity in pups with maternal BPA exposure. Therefore, we focused on the hippocampus, which is the part of the central nervous system that is thought to be most strongly affected by BPA, as shown by recent studies, and performed MSD–AFLP analysis which is a new method that can detect genome-wide CpG methylation level changes with a difference in the methylation level of less than 5% and a variation rate of the methylation level of less than 10% in an inter-tissue comparison experiment [39]. However, in this study, no statistically significant differences in methylation level between the control group and the BPA-exposed group were detected in all CpGs (43,840) analyzed in the hippocampus.

The effects of BPA exposure on CpG methylation level in genomic DNA in the brain and gonads have been reported. BPA at a low dose (20 μg/kg) was administered to pregnant ICR/Jcl mice and the methylation level changes in the forebrain of the fetus was analyzed by the genome-wide restriction landmark genomic scanning (RLGS) method [37], which showed that the methylation levels of 48 CpGs were altered. A low dose BPA (10 μg/kg) injection to pups of Sprague-Dawley (SD) rat has also been reported to lead to the hypomethylation of the phosphodiesterase type 4 gene in the prostate and increase susceptibility to 7,12-dimethylbenz[a]anthracene (DMBA)-induced prostate carcinogenesis [35]. On the other hand, in utero and lactational exposure of BPA (0.05, 7.5, 30 and 120 mg/kg/day, by oral) to the mother rats of inbred Fisher 344 strain did not result in the DMBA-induced carcinogenesis of the prostate [40]. The urinary excretion rate of BPA were 21% and 70%, and the fecal excretion rates of BPA were 42% and 50% in SD and Fisher 344 rats, respectively [41]. Furthermore, the absorption, distribution, metabolic rate, and excretion rate (ADME) vary depending on the rat strain. Therefore, differences in ADME values among strains may be reflected in blood BPA level and may have affected the experimental results. Additionally, there is an argument that closed colonies such as ICR mice and SD rats show genetic diversity within colonies, resulting in false positives data that occasionally occur due to genetic diversity that may arise even by simply dividing pups into two groups [42]. Thus, it is preferable to use inbred strain animals to exclude genetic variation among individuals in the analysis of epigenomic changes.

As an example, when using inbred *Avy* mice, it has been reported that prenatal BPA exposure (50-mg BPA/kg diet) decreased the CpG methylation levels of the transposon sequence in *Agouti* by about 10% [33]. *Agouti* determines body hair color and the inserted transposon methylation negatively regulates gene expression. Therefore, they concluded that the yellow hair of *Avy* mice changed into brown due to this hypomethylation. However, they did not study whether there was any alteration in other genes. Using inbred BALB/c mice, there was a report that prenatal exposure of BPA (200 μg/kg/day, by oral) hypermethylated the transcriptional regulatory region of the hippocampal neurotrophic factor (BDNF) of the males and consequently decreased the mRNA expression level [36]. Additionally, they also analyzed using free DNA in blood and interestingly showed that brain BDNF methylation levels correlate with those of blood DNA [36]. The levels of 17β-estradiol and testosterone and the ratio of these two sex hormones were different between mouse strains [43]. The difference in the endocrine system between C57BL/6 J and CBA/Lac mice appears to affect the activity of endocrine disruptors, such as BPA. In addition, although 17β-estradiol had been used to positive control in the studies of effect for prenatal BPA exposure of SD rat offspring on increase DMBA-induced prostate carcinogenesis [35], neonatal exposure of 17β-estradiol had no effects on mutagenicity of DMBA in reproductive tissues of adult Big Blue transgenic mice [44]. Thus, the difference in genetic background may change the effects of BPA. Although we analyzed 43,840 CpGs in this study, which are approximately 0.3% out of the total 20,000,000 CpGs in mouse genome, we were unable to detect statistically significant changes in CpG methylation levels, which might be due to the stability of the methylated CpG in the C57BL/6 J mouse hippocampus against small amount of environmental chemicals. If further analysis is performed with a wider coverage, it may be possible to detect significant BPA-specific methylation level alterations even in the C57BL/6 J hippocampus.

## Conclusions

In this study, we investigated whether BPA alters CpG methylation levels in the mouse hippocampus. Despite the use of MSD–AFLP analysis, which is a high-precision and highly sensitive analytical method, no changes in methylation levels as the effect of low-dose BPA exposure were detected. Therefore, although further analysis is necessary, we concluded that under the experimental conditions of the present study, the effects of prenatal BPA exposure on the hippocampal DNA methylation are extremely small.

## Additional file

Additional file 1: **Table S1**. The pup number and sex ratio. **Figure S1**. Effect of prenatal BPA exposure on body weight and anogenital distance (AGD). **Figure S2**. A typical MSD-AFLP peak chart after electrophoresis obtained by a selective primer set. **Figure S3**. MSRE-PCR analysis of representative CpGs (Chr 4: 35339023 and Chr X: 74707008) showing the minimum q-value obtained from the MSD-AFLP data. **Table S2**. The KEGG enrichment analysis of the effect on the DNA methylation. (DOCX 289 kb)

## Abbreviations

AGD: Anogenital distance
BPA: Bisphenol A
DOHaD: Developmental Origins of Health and Disease
FDR: False discovery rate
GFDB: Genome DNA Fragment Database
HCA: Hierarchical clustering analysis
KEGG: Kyoto Encyclopedia Genes and Genome
MSD-AFLP: Methylated site display-amplified fragment length polymorphism
MSRE-PCR: Methylation-sensitive restriction enzyme-dependent PCR
PCA: Principal component analysis
UGC: Urogenital complex
