# Supplementary material for: Does the prenatal bisphenol A exposure alter DNA methylation levels in the mouse hippocampus?: An analysis using a high-sensitivity methylome technique
Source: Genes Environ. 2018 Jun 4;40:12. doi: 10.1186/s41021-018-0099-y (PMC5985587; doi:10.1186/s41021-018-0099-y)
Supplement: Supplementary file 1 — Table S1. The pup number and sex ratio. Figure S1. Effect of prenatal BPA exposure on body weight and anogenital distance (AGD). Figure S2. A typical MSD-AFLP peak chart after electrophoresis obtained by a selective primer set. Figure S3. MSRE-PCR analysis of representative CpGs (Chr 4: 35339023 and Chr X: 74707008) showing the minimum q-value obtained from the MSD-AFLP data. Table S2. The KEGG enrichment analysis of the effect on the DNA methylation. (DOCX 289 kb) [file 41021_2018_99_MOESM1_ESM.docx]

**Table S1. The pup number and sex ratio.**

|  | **Dam** | **Male** | **Female** | **Total** |
| --- | --- | --- | --- | --- |
| Control | 5 | 24 | 22 | 47 |
| BPA | 6 | 29 | 31 | 60 |


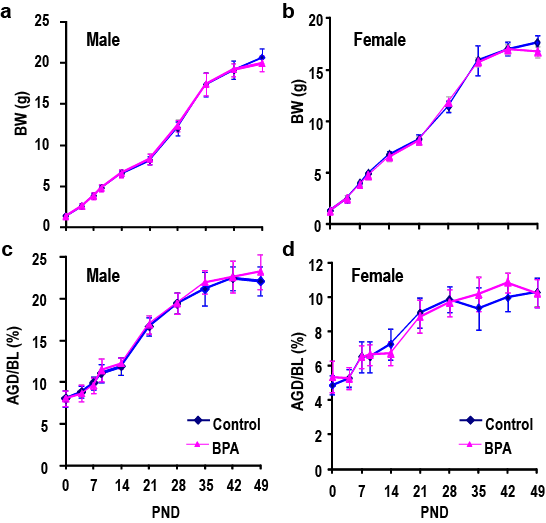


**Figure S1**. Effect of prenatal BPA exposure on body weight and anogenital distance (AGD). (a) Body weight change from birth through PND 49 of male mice (a) and female mice (b). AGD normalized by body length (BL) of male mice (c) and female mice (d).

**
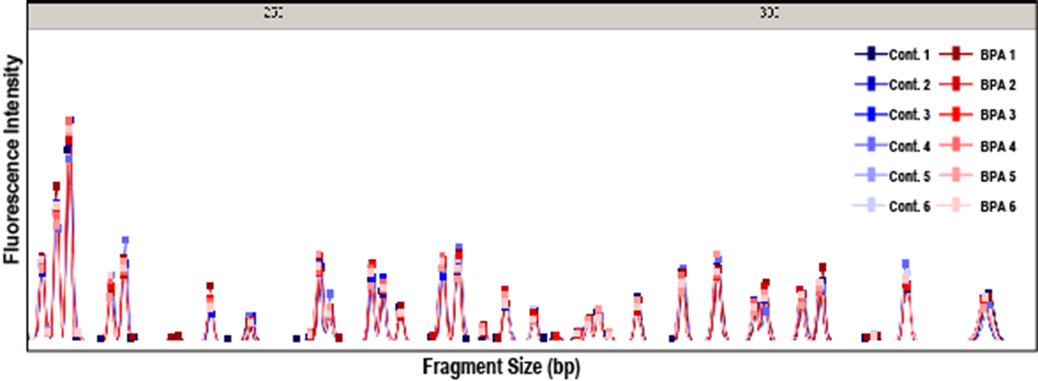
**

**Figure S2**. A typical MSD-AFLP peak chart after electrophoresis obtained by a selective primer set. Twelve electropherograms are seen on the chart (Control, 6 mice; BPA-treated, 6 mice).


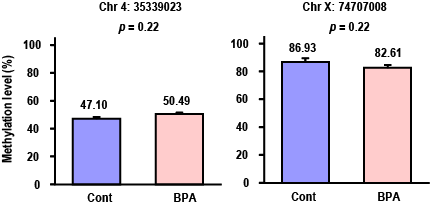


**Figure S3**. MSRE-PCR analysis of representative CpGs (Chr 4: 35339023 and Chr X: 74707008) showing the minimum q-value obtained from the MSD-AFLP data. There was no statistical significance in methylation levels of the two CpGs.

**Table S2. The KEGG enrichment analysis of the effect on the DNA methylation.**

| **BPA > Control** | | | |  | **BPA < Control** | | | |
| --- | --- | --- | --- | --- | --- | --- | --- | --- |
| **KEGG pathway** | **SIZE** | **NES** | **FDR** |  | **KEGG pathway** | **SIZE** | **NES** | **FDR** |
| N-Glycan Biosynthesis | 12 | 1.600 | 0.776 |  | Histidine Metabolism | 7 | -1.602 | 0.917 |
| Fc Epsilon Ri Signaling Pathway | 22 | 1.450 | 1.000 |  | Alzheimers Disease | 36 | -1.533 | 0.953 |
| T Cell Receptor Signaling Pathway | 28 | 1.443 | 1.000 |  | Dna Replication | 6 | -1.433 | 1.000 |
| Aminoacyl Trna Biosynthesis | 8 | 1.397 | 1.000 |  | Ecm Receptor Interaction | 36 | -1.419 | 1.000 |
| Mtor Signaling Pathway | 14 | 1.376 | 1.000 |  | Fatty Acid Metabolism | 5 | -1.415 | 1.000 |
| B Cell Receptor Signaling Pathway | 24 | 1.336 | 1.000 |  | Pancreatic Cancer | 22 | -1.411 | 0.870 |
| Ubiquitin Mediated Proteolysis | 25 | 1.317 | 1.000 |  | Nucleotide Excision Repair | 8 | -1.372 | 0.957 |
| Purine Metabolism | 49 | 1.253 | 1.000 |  | Parkinsons Disease | 14 | -1.340 | 1.000 |
| Viral Myocarditis | 17 | 1.247 | 1.000 |  | Pathways In Cancer | 112 | -1.291 | 1.000 |
| Tight Junction | 45 | 1.235 | 1.000 |  | Maturity Onset Diabetes Of The Young | 6 | -1.282 | 1.000 |
| Vasopressin Regulated Water Reabsorption | 15 | 1.233 | 1.000 |  | Antigen Processing And Presentation | 5 | -1.275 | 1.000 |
| Type I Diabetes Mellitus | 5 | 1.226 | 1.000 |  | Arginine And Proline Metabolism | 10 | -1.268 | 1.000 |
| Glycerophospholipid Metabolism | 20 | 1.221 | 1.000 |  | Porphyrin And Chlorophyll Metabolism | 7 | -1.224 | 1.000 |
| O-Glycan Biosynthesis | 11 | 1.221 | 0.966 |  | Arrhythmogenic Right Ventricular Cardiomyopathy Arvc | 39 | -1.201 | 1.000 |
| Spliceosome | 13 | 1.218 | 0.916 |  | Bladder Cancer | 12 | -1.198 | 1.000 |
| Circadian Rhythm Mammal | 5 | 1.216 | 0.866 |  | Tyrosine Metabolism | 5 | -1.198 | 1.000 |
| Ether Lipid Metabolism | 9 | 1.193 | 0.908 |  | Valine Leucine And Isoleucine Degradation | 11 | -1.194 | 1.000 |
| Alanine Aspartate And Glutamate Metabolism | 7 | 1.184 | 0.895 |  | Wnt Signaling Pathway | 53 | -1.169 | 1.000 |
| Leukocyte Transendothelial Migration | 31 | 1.180 | 0.862 |  | Amyotrophic Lateral Sclerosis Als | 19 | -1.162 | 1.000 |
| Snare Interactions In Vesicular Transport | 10 | 1.170 | 0.860 |  | Tgf Beta Signaling Pathway | 15 | -1.159 | 1.000 |
| Leishmania Infection | 7 | 1.160 | 0.850 |  | Gap Junction | 37 | -1.148 | 1.000 |
| Type II Diabetes Mellitus | 16 | 1.158 | 0.820 |  | Basal Transcription Factors | 7 | -1.147 | 0.977 |
| Glycerolipid Metabolism | 14 | 1.137 | 0.852 |  | Drug Metabolism Other Enzymes | 7 | -1.147 | 0.936 |
| Inositol Phosphate Metabolism | 21 | 1.132 | 0.835 |  | Colorectal Cancer | 21 | -1.141 | 0.914 |
| Taste Transduction | 15 | 1.122 | 0.832 |  | Prion Diseases | 8 | -1.132 | 0.910 |
| Fc Gamma R Mediated Phagocytosis | 32 | 1.117 | 0.815 |  | Oxidative Phosphorylation | 11 | -1.131 | 0.879 |
| Dorso Ventral Axis Formation | 9 | 1.113 | 0.797 |  | Cell Cycle | 23 | -1.111 | 0.915 |
| Regulation Of Autophagy | 7 | 1.101 | 0.806 |  | P53 Signaling Pathway | 17 | -1.109 | 0.889 |
| Phosphatidylinositol Signaling System | 30 | 1.099 | 0.783 |  | Tryptophan Metabolism | 7 | -1.102 | 0.876 |
| Nicotinate And Nicotinamide Metabolism | 6 | 1.090 | 0.781 |  | Adipocytokine Signaling Pathway | 19 | -1.101 | 0.850 |
| Small Cell Lung Cancer | 32 | 1.087 | 0.763 |  | Regulation Of Actin Cytoskeleton | 75 | -1.100 | 0.824 |
| Calcium Signaling Pathway | 64 | 1.083 | 0.752 |  | Amino Sugar And Nucleotide Sugar Metabolism | 9 | -1.096 | 0.812 |
| Sphingolipid Metabolism | 9 | 1.077 | 0.745 |  | Focal Adhesion | 78 | -1.082 | 0.829 |
| Acute Myeloid Leukemia | 19 | 1.023 | 0.869 |  | Mismatch Repair | 5 | -1.076 | 0.822 |
| Progesterone Mediated Oocyte Maturation | 24 | 1.017 | 0.861 |  | Glutathione Metabolism | 9 | -1.072 | 0.812 |
| Glycosphingolipid Biosynthesis Lacto And Neolacto Series | 6 | 1.014 | 0.846 |  | Cytokine Cytokine Receptor Interaction | 44 | -1.048 | 0.856 |
| Rig I Like Receptor Signaling Pathway | 10 | 1.008 | 0.839 |  | Melanoma | 23 | -1.031 | 0.885 |
| Peroxisome | 16 | 0.964 | 0.933 |  | Dilated Cardiomyopathy | 45 | -1.028 | 0.869 |
| Prostate Cancer | 33 | 0.956 | 0.929 |  | Chronic Myeloid Leukemia | 22 | -1.012 | 0.896 |
| Erbb Signaling Pathway | 31 | 0.952 | 0.915 |  | Vascular Smooth Muscle Contraction | 43 | -0.995 | 0.921 |
| Ppar Signaling Pathway | 14 | 0.951 | 0.896 |  | Olfactory Transduction | 8 | -0.994 | 0.901 |
| Vibrio Cholerae Infection | 14 | 0.936 | 0.910 |  | Notch Signaling Pathway | 18 | -0.993 | 0.881 |
| Butanoate Metabolism | 8 | 0.930 | 0.905 |  | Basal Cell Carcinoma | 20 | -0.991 | 0.867 |
| Axon Guidance | 56 | 0.925 | 0.896 |  | Arachidonic Acid Metabolism | 11 | -0.991 | 0.848 |
| Non Small Cell Lung Cancer | 18 | 0.890 | 0.957 |  | Hypertrophic Cardiomyopathy Hcm | 39 | -0.984 | 0.845 |
| Chemokine Signaling Pathway | 59 | 0.874 | 0.975 |  | Intestinal Immune Network For Iga Production | 8 | -0.970 | 0.864 |
| Thyroid Cancer | 10 | 0.853 | 1.000 |  | Pathogenic Escherichia Coli Infection | 9 | -0.960 | 0.869 |
| Complement And Coagulation Cascades | 11 | 0.850 | 0.986 |  | Lysine Degradation | 10 | -0.960 | 0.853 |
| Cysteine And Methionine Metabolism | 6 | 0.848 | 0.970 |  | Hedgehog Signaling Pathway | 19 | -0.957 | 0.841 |
| Endometrial Cancer | 18 | 0.846 | 0.956 |  | Mapk Signaling Pathway | 94 | -0.932 | 0.883 |
| Toll Like Receptor Signaling Pathway | 19 | 0.835 | 0.960 |  | Natural Killer Cell Mediated Cytotoxicity | 27 | -0.926 | 0.879 |
| Lysosome | 41 | 0.829 | 0.955 |  | Long Term Depression | 29 | -0.922 | 0.873 |
| Vegf Signaling Pathway | 26 | 0.826 | 0.941 |  | Pyrimidine Metabolism | 21 | -0.905 | 0.896 |
| Endocytosis | 60 | 0.806 | 0.960 |  | Cardiac Muscle Contraction | 18 | -0.894 | 0.905 |
| Jak Stat Signaling Pathway | 26 | 0.791 | 0.970 |  | Fructose And Mannose Metabolism | 6 | -0.875 | 0.929 |
| Glioma | 25 | 0.778 | 0.974 |  | Oocyte Meiosis | 28 | -0.844 | 0.981 |
| Long Term Potentiation | 28 | 0.767 | 0.976 |  | Neurotrophin Signaling Pathway | 42 | -0.842 | 0.967 |
| Citrate Cycle Tca Cycle | 5 | 0.766 | 0.960 |  | Nod Like Receptor Signaling Pathway | 5 | -0.841 | 0.953 |
| Homologous Recombination | 7 | 0.749 | 0.969 |  | Rna Degradation | 15 | -0.833 | 0.951 |
| Galactose Metabolism | 7 | 0.736 | 0.972 |  | Adherens Junction | 39 | -0.832 | 0.937 |
| Insulin Signaling Pathway | 38 | 0.735 | 0.957 |  | Ribosome | 5 | -0.826 | 0.934 |
| Selenoamino Acid Metabolism | 5 | 0.713 | 0.970 |  | Cytosolic Dna Sensing Pathway | 5 | -0.822 | 0.926 |
| Glycosphingolipid Biosynthesis Ganglio Series | 6 | 0.712 | 0.956 |  | Pyruvate Metabolism | 11 | -0.818 | 0.919 |
| Glycosaminoglycan Degradation | 8 | 0.671 | 0.981 |  | Renal Cell Carcinoma | 23 | -0.805 | 0.927 |
| Propanoate Metabolism | 10 | 0.659 | 0.978 |  | Cell Adhesion Molecules Cams | 37 | -0.772 | 0.968 |
| Apoptosis | 24 | 0.584 | 1.000 |  | Gnrh Signaling Pathway | 40 | -0.748 | 0.991 |
| Primary Immunodeficiency | 5 | 0.583 | 0.995 |  | Huntingtons Disease | 30 | -0.734 | 0.997 |
| Metabolism Of Xenobiotics By Cytochrome P450 | 8 | 0.572 | 0.985 |  | Systemic Lupus Erythematosus | 10 | -0.732 | 0.985 |

NES, normalized enrichment score; FDR, false discovery rate *q*-value.
